# Robustness of Cyber-Physical Supply Networks in Cascading Failures

**DOI:** 10.3390/e23060769

**Published:** 2021-06-18

**Authors:** Dong Mu, Xiongping Yue, Huanyu Ren

**Affiliations:** School of Economics and Management, Beijing Jiaotong University, Beijing 100044, China; dmu@bjtu.edu.cn (D.M.); 19113044@bjtu.edu.cn (H.R.)

**Keywords:** robustness, cascading failure, cyber-physical supply networks, underload, overload

## Abstract

A cyber-physical supply network is composed of an undirected cyber supply network and a directed physical supply network. Such interdependence among firms increases efficiency but creates more vulnerabilities. The adverse effects of any failure can be amplified and propagated throughout the network. This paper aimed at investigating the robustness of the cyber-physical supply network against cascading failures. Considering that the cascading failure is triggered by overloading in the cyber supply network and is provoked by underload in the physical supply network, a realistic cascading model for cyber-physical supply networks is proposed. We conducted a numerical simulation under cyber node and physical node failure with varying parameters. The simulation results demonstrated that there are critical thresholds for both firm’s capacities, which can determine whether capacity expansion is helpful; there is also a cascade window for network load distribution, which can determine the cascading failures occurrence and scale. Our work may be beneficial for developing cascade control and defense strategies in cyber-physical supply networks.

## 1. Introduction

The physical and digital worlds are becoming continuously more intertwined, bringing about cyber-physical supply networks with emergent interactions [1]. The physical supply network depends on the cyber supply network for its control, and the cyber supply network depends on the physical network for flow information. While the management of cyber-physical supply networks is a challenging problem [2], these interdependent systems tend to be fragile against failures, hazards, and attacks [3]. Due to functional interdependency, a failure of firms in one network results in a failure of dependent firms in other networks, which may induce further damage to the first network and so on [4]. The failures can trigger multiple parts of supply networks, influencing the performance and viability of the components of the entire network [5].

The cyber-physical supply network can be modeled as an interdependent network to indicate the complex interdependencies of its subsystems and components [5,6]. The subsystems and components can be signified as nodes, and the dependencies can be represented as links. For example, nodes can denote firms in the physical supply network, and links can denote conveyance mechanisms [7]. With the context of CPS and complex networks, failure can be defined as a form of deadlock, where all firms of the network are halted while waiting for products, and the complex interdependencies between the firms of the network make freeing the deadlock difficult [8,9]. A cascading failure process is one in which the failure of one or more properties in a network (links/nodes) can trigger the failure of other parts of the network [10]. 

Overloading failure will prevent the transmission of data package information and lead to a decrease in the efficiency of the cyber supply network [11]. The overload phenomenon implies that data flow exceeds the node’s capacity in the cyber supply network. In this case, data will be transmitted by the node closest to the affected node with sufficient capacity [12]. If the adjacent load does not exceed the capacity, the cyber supply network usually operates. If not, the adjacent nodes will overload, leading to further redistribution of load and the accumulation of cascading failures. 

Unlike overload failures, failures of firms in the physical supply network result from underloading. When firms cannot fulfill the expected production requirement to overcome the fixed production costs, they will fail to gain profit and possibly exit the market [13]. A firm’s failure can decrease the product demand from upstream firms, which may force the upstream firms to stop production. Moreover, a firm’s failure can decrease material supply for downstream firms, which may force downstream firms to shut down. For example, during the COVID-19 pandemic, the inability of suppliers to provide a diverse set of resources to complex networks of organizations led to large parts of the supply chain becoming deadlocked [8]. Therefore, an underload cascading failure model is more suitable for the physical supply network [14].

The robustness of the cyber-physical supply network is usually defined as the relative size of the firms that survive the cascading failures [11]. Our goal was to construct a cascading failure model that can quantify the robustness of the cyber-physical supply network to provide a scientific basis for the development of network protection. Considering that the cascading failure is triggered by overloading in the cyber supply network and provoked by underloading in the physical supply network, this paper tried to answer the following questions: 

RQ1: How can we find a certain region of the parameter space where cascade failures occur under cyber node failure?

RQ2: How can we find a certain region of the parameter space where cascade failures occur under physical node failure?

This study makes two main contributions. First, unlike the traditional analysis on the overload cascading failure model, this study explored overloading in the cyber supply network and underloading in the physical supply network. Second, this study uncovered the cascade window for cyber-physical supply networks. The parameter space can determine the occurrence and scale of the cascading failure. The rest of this article is organized as follows. Section 2 reviews the literature on cyber-physical supply networks in cascading failures. Section 3 introduces the model for cyber-physical supply networks. Section 4 presents the cascading failure model in cyber-physical supply networks. Section 5 describes numerical simulation. Finally, Section 6 draws the discussion and conclusion.

## 2. Literature Review

In this section, the relevant literature is discussed and classified. This review is intended to offer an overview of recent studies surrounding failure, cascading failure, cyber supply networks, physical supply networks, and robustness metrics. The review of the related works is summarized in Table 1.

In a supply network, a firm’s operation is usually influenced by its upstream and downstream firms, and the failure of any firm (node) could cause the whole network to fail [14]. Such failure may delay the flow of goods, information, and funds in supply networks and affect the normal operations of many other firms due to cascading failure [20]. The indirect effects of production failures due to propagation are substantially larger than their direct effects [21]. Adding only single links may undermine normal supply network operation and stimulate disturbances remotely from the location of the structural change, and even cause global cascades of failures [22]. 

The phenomena of cascading failures often occur in complex networks, where the node failures can trigger overloading and underloading. Overloading may cause further failures of neighbor nodes and, finally, cascading failures of the global network. In [18], an extended cascading failure process triggered by resource/load fluctuations was proposed, considering the overload of the supply nodes and resource deficiency of the demand nodes. The load is preferentially redistributed along those higher-capacity nodes attached to the failed node [20]. Unlike overload failures, firms’ subsequent failures in supply networks result from underloading [14]. For the underload cascading failure model without a recovery process, a discontinuous phase transition was found [13]. The size of cascades of underload failures is related to the lower limit of node capacity [19].

A supply network is a distinct interdependent network composed of a cyber supply network and a physical supply network [15]; additionally, [23] proposed that supply chain models integrate physical and cyber networks. In supply chain systems, the communication comprises the entity’s supplier, manufacturers, and distribution centers, which can acquire the data of demand, stock, and production [2]. Intertwining the virtual supply chain with the physical supply chain and their operations makes the additive manufacturing process a cyber-physical system [24]. The author of [25] introduced the structure dynamics control concept and a dynamic model to orchestrate operations in cyber-physical supply chains in smart manufacturing. A cyber-physical e-commerce logistics system has been applied in Hong Kong. In the physical world, industrial wearable technology transforms assets into cloud assets. In the cyber world, synchronization mechanisms enhance the utilization ratio of resources and spaces while decreasing waiting and wastage [26].

Several robustness metrics have been developed to compute the damage caused by cascading failures. The author of [3] defined the giant mutually connected component as the mutually connected cluster spanning the entire network. The author of [15] used a comprehensive effectiveness index to represent the average robustness of an interdependent supply network. The robustness is also quantified as the surviving fraction of nodes at the end of cascading failures [17]. The concept of network efficiency can quantify the consequence of cascading failures in the supply network [14]. 

The literature survey observed that most related works have not specifically investigated the robustness of cyber-physical supply networks in overload and underload cascading failures. The existing model mainly considers the impact of overload on the cascading process of supply networks but ignores the impact of underload. The fact that supply networks consist of the cyber supply network and physical supply network is often unconsidered. Moreover, the reality that the cascading failure is triggered by overload in the cyber supply network and provoked by underload in the physical supply network is usually ignored. This knowledge gap is addressed by the overload and underload cascading failures model discussed in the following section.

## 3. The Theoretical Model of Cyber-Physical Supply Networks

This section is divided by subheadings and provides a precise description of the experimental results, their interpretation, and the experimental conclusions that can be drawn.

**Figure 1:**
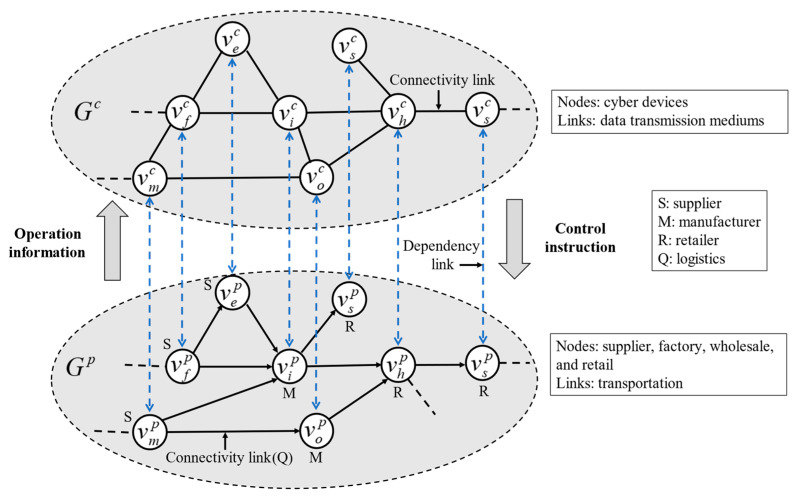
System model illustration for the cyber-physical supply networks, where network $G^{p}$ is the physical supply network, and network $G^{c}$ is the cyber supply network. Interdependence across the two networks is realized by the one-to-one support links shown by dash lines.

As the interdependencies between the physical supply network and cyber supply network give rise to multiple possible failure spreading channels, a firm’s failure can rashly influence its associated predecessor and successor firms and initiate a cascade of firm failures that can imperil the supply networks’ operation. The load-capacity model in interdependent supply networks is built to duplicate the catastrophic propagation process. A comprehensive description of the proposed cyber-physical supply networks is presented in this section, as shown in Figure 1. The symbols used in the model are explained in Table 2.

### 3.1. Cyber Supply Network

#### 3.1.1. The Nodes and Links of the Cyber Supply Network

The cyber supply network comprises various functional cyber devices that generate, store, transform, receive, and transmit signals or information [27]. In the cyber supply network, all functional cyber devices are denoted as nodes, and the data transmission mediums between devices are links. Therefore, the cyber supply network as a weighted undirected network is $G^{c}(V^{c},E^{c})$, where $V^{c}=(v_{1}^{c},v_{2}^{c},...,v_{N}^{c})$ is the node set and $E^{c}=\left\{\left(v_{i}^{c},v_{j}^{c}\right)|e_{ij}^{c}=0\;or\;1,i,j=1,2,3,...,n\right\}$ is the set of connectivity links. Here, $e_{ij}^{c}=1$ signifies a connection from node $v_{i}^{c}$ to node $v_{j}^{c}$; otherwise, $e_{ij}^{c}=0$. Further, $W^{p}$ is constructed to represent the flow constraints of the links, where $W^{c}=[w_{ij}^{c}]$ is an N×N asymmetric matrix and N is the total number of nodes in the cyber supply network. The weight can be defined as:

(1) $$w_{ij}^{c}={\left(k_{v_{i}^{c}}*k_{v_{j}^{c}}\right)}^{\tau },\;$$

where τ is the weight parameter of the link in the cyber supply network.

#### 3.1.2. Load and Capacity of the Cyber Supply Network

Each node $v_{i}^{c}$ generates the same number of packets per second and can also be involved in a router in the meantime. The routing protocol makes each packet go through the shortest path to make the cyber supply network efficient. In this way, we define the initial load of cyber node $v_{i}^{c}$ to be

(2) $$L_{v_{i}^{c}}=(\sum_{v_{k}^{c}\neq v_{i}^{c}\neq v_{j}^{c}\in G^{c}}\frac{\sigma_{v_{k}^{c}v_{j}^{c}}(v_{i}^{c})}{\sigma_{v_{k}^{c}v_{j}^{c}}}{)}^{\delta },\;$$

where $\sigma_{v_{k}^{c}v_{j}^{c}}$ signifies the number of shortest paths from $v_{k}^{c}$ to $v_{j}^{c}$, $\sigma_{v_{k}^{c}v_{j}^{c}}(v_{i}^{c})$ is the number of shortest paths from $v_{k}^{c}$ to $v_{j}^{c}$ that go through node $v_{i}^{c}$, and δ is the tunable parameter used to dominate the strength of the initial node load. We define the maximum amount of flow that node $v_{i}^{c}$ can process as its capacity and assume the this is proportional to its initial load,

(3) $$C_{v_{i}^{c}}(max)=\varphi L_{v_{i}^{c}}(0),\;$$

where φ(φ>1) is the upper-bound parameter of the node and $L_{v_{i}^{c}}(0)$ is the initial load of node $v_{i}^{c}$.

### 3.2. Physical Supply Network

#### 3.2.1. Nodes and Links of the Physical Supply Network

The physical supply network consists of suppliers, manufacturers, retailers, and logistics that generate, store, transform, and deliver the flow of physical products [28], all of which can be signified as nodes, and the contractual relationship between firms can be signified as links [12,15]. Therefore, the physical supply network as a weighted directed network is $G^{p}(V^{p},E^{p})$, where $V^{p}=\left\{v_{1}^{p},v_{2}^{p},...,v_{n}^{p}\right\}$ is the node set and $E^{p}=\left\{\left(v_{i}^{p},v_{j}^{p}\right)|e_{ij}^{p}=0\;or\;1,i,j=1,2,3,...,n\right\}$ is the set of connectivity links. Here, $e_{ij}^{p}=1$ signifies a directed connection from node $v_{i}^{p}$ to node $v_{j}^{p}$; otherwise, $e_{ij}^{p}=0$. A weighted adjacency matrix $W^{p}$ is constructed to represent the weights of the links, where $W^{p}=[w_{ij}^{p}]$ is an N×N asymmetric matrix and N is the total number of nodes in the physical supply network. The links between the nodes with greater values of tend to have a long distance.

The transportations between firms are regarded as the weights of the links. The empirical studies have proposed that the weight of the links between two nodes is related to the node’s degree [14]. Therefore, the weight of a link $w_{ij}^{p}$ that connects $v_{i}^{p}$ to $v_{j}^{p}$ is assumed to be:

(4) $$w_{ij}^{p}={\left(k_{v_{i}^{p}}*k_{v_{j}^{p}}\right)}^{\theta },\;$$

where θ is the weight parameter of the link in the physical supply network, $k_{v_{i}^{p}}$ indicates the degree for $v_{i}^{p}$, and $k_{v_{i}^{p}}=k_{v_{i}^{p}}^{in}+k_{v_{i}^{p}}^{out}$. $k_{v_{i}^{p}}^{in}$ is the in-degree of node $v_{i}^{p}$ and $k_{v_{i}^{p}}^{out}$ is the out-degree of node $v_{i}^{p}$. The degree of node $v_{i}^{p}$ is represented as:

(5) $$k_{v_{i}^{p}}=\sum_{j\in V^{p}}e_{ij}^{p}$$

#### 3.2.2. Load and Capacity of the Physical Supply Network

The material flows can be treated as the loads in the physical supply network. Specifically, material flows describe the transport of material, components, or products [14,29]. Three methods are used to signify the node’s initial load, which include the node degree centrality [30], node betweenness centrality [31], or the node-outdegree centrality [15]. As the operation of firms in the physical supply network is related to both upstream firms and downstream firms, the initial load $L_{v_{i}^{p}}(0)$ for $v_{i}^{p}$ is defined as a function of the degree of $v_{i}^{p}$:

(6) $$L_{v_{i}^{p}}(0)={\left(k_{v_{i}^{p}}\right)}^{\alpha },\;$$

where α is the tunable parameter used to adjust the initial load.

The physical supply network often transfers some loads, where the most massive load that a node can deal with is named the capacity. A node’s capacity is limited due to confined cost. For example, the supply capacity and manufacturing capacity of a firm are restricted by the firm’s scale. In other words, each node has a specific upper-bound capacity, which is linearly correlated with its initial load. The upper node capacity $C_{v_{i}^{p}}(max)$ is

(7) $$C_{v_{i}^{p}}(max)=\beta L_{v_{i}^{p}}(0),\;$$

where β(β>1) is the upper-bound parameter of the node.

Furthermore, the physical supply network works to provide products and services to customers, and the goal of each firm is to obtain revenue. If a firm’s product demand or raw material supply is below a certain level, the company will not operate normally and eventually close down due to unprofitability. Therefore, the load to maintain the firm’s regular operation must be higher than a specific limit. The lower-bound capacity $C_{v_{i}^{p}}(min)$ for $v_{i}^{p}$ is presented as follows:

(8) $$C_{v_{i}^{p}}(min)=\gamma L_{v_{i}^{p}}(0),\;$$

where γ(0<γ<1) is the lower-bound parameter of the node.

### 3.3. Description of Interdependence Relations

In cyber-physical supply networks, the cyber supply network and physical supply network mutually coordinate to attain dynamic closed-loop control. On the one hand, the firms in the physical supply network, such as suppliers and manufacturers, should provide flow information to devices in the cyber supply network for analysis. On the other hand, cyber supply network devices need to provide commands to firms in the physical supply network for control. Therefore, the two networks are assumed to display one-to-one interdependence. This coupling relation ensures that each node in the physical supply network has only one support node in the cyber supply network and vice versa [32]. Moreover, it should be noted that the topological structures of the two subsystems are not necessarily identical [33]. Therefore, let $E_{ik}^{pc}$ be a set of dependency links connecting nodes between network $G^{p}$ and network $G^{c}$; then, M is defined as $M=\left\{E_{ik}^{pc}\subseteq v_{i}^{p}\times v_{k}^{c};\;v_{i}^{p}\in V^{p},v_{k}^{c}\in V^{c}\right\}$. The dependency link $(v_{i}^{p},v_{k}^{c})\in E_{ik}^{pc}$ represents node $v_{i}^{p}$ depending on node $v_{k}^{c}$, and vice versa.

## 4. Modeling Cascading Failures in Cyber-Physical Supply Networks

### 4.1. Cascading Failure Model

In the cyber-physical supply networks, random failures are high impact, low probability events, whereas targeted failures are low impact, high probability events [34]. Targeted failures can be reduced by controlling associated risks such as forecast inaccuracy, quality, and production system breakdown. However, random failures are very hard to control because they are triggered by uncontrollable factors [35]. Therefore, this study concentrated on the random failures caused by nature, the political system, and available capacity. If a node $v_{i}^{p}$ in the physical supply network fails, it will influence its neighboring nodes and the dependent nodes in the cyber supply network, which may cause further failure to the physical supply network. The dependency links between the interdependent network play the role of connection and do not receive the redistributed load of the failed node, so the redistribution of load flows only on the intralayer network.

#### 4.1.1. Underload Cascading Failure in the Physical Supply Network

**Figure 2:**
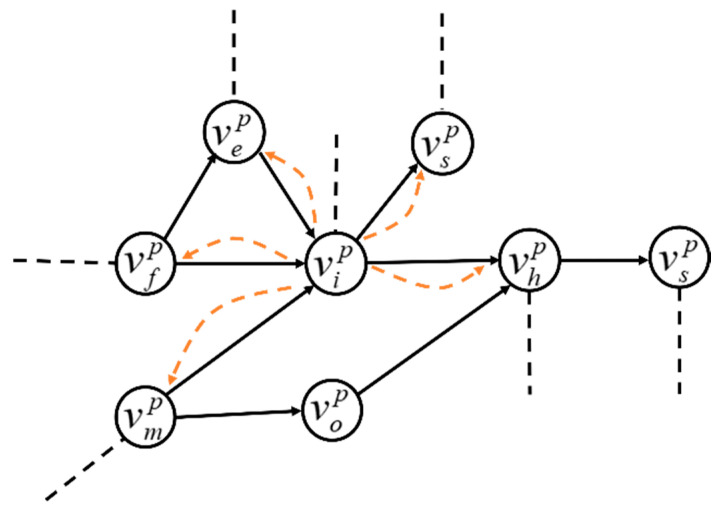
Schematic diagram of load redistribution after a node failure.

When a firm fails, it can neither receive supplies from upstream neighbors nor ship products to its downstream customers [13]. As shown in Figure 2, when the failure occurs on a node $v_{i}^{p}$, its upstream and downstream nodes with contractual relationships are impacted. The reduced loads of the upstream and downstream nodes nearby $v_{i}^{p}$ are calculated as

(9) $$\left\{ \begin{array}{l} \Delta L_{is}^{p}{}^{-}=L_{i}^{p}(0)\frac{w_{is}}{\sum_{g\in \Gamma_{i}^{in}}w_{gi}+\sum_{g^{\prime }\in \Gamma_{i}^{out}}w_{ig^{\prime }}}\\ \Delta L_{ih}^{p}{}^{-}=L_{i}^{p}(0)\frac{w_{ih}}{\sum_{g\in \Gamma_{i}^{in}}w_{gi}+\sum_{g^{\prime }\in \Gamma_{i}^{out}}w_{ig^{\prime }}}\\ \Delta L_{ei}^{p}{}^{-}=L_{i}^{p}(0)\frac{w_{ei}}{\sum_{g\in \Gamma_{i}^{in}}w_{gi}+\sum_{g^{\prime }\in \Gamma_{i}^{out}}w_{ig^{\prime }}}\\ \Delta L_{fi}^{p}{}^{-}=L_{i}^{p}(0)\frac{w_{fi}}{\sum_{g\in \Gamma_{i}^{in}}w_{gi}+\sum_{g^{\prime }\in \Gamma_{i}^{out}}w_{ig^{\prime }}}\\ \Delta L_{mi}^{p}{}^{-}=L_{i}^{p}(0)\frac{w_{mi}}{\sum_{g\in \Gamma_{i}^{in}}w_{gi}+\sum_{g^{\prime }\in \Gamma_{i}^{out}}w_{ig^{\prime }}} \end{array}\right.$$

where $\Delta L_{is}^{p}{}^{-}$, $\Delta L_{ih}^{p}{}^{-}$, $\Delta L_{ei}^{p}{}^{-}$, $\Delta L_{fi}^{p}{}^{-}$, and $\Delta L_{mi}^{p}{}^{-}$ are the reduced load for upstream and downstream nodes near $v_{i}^{p}$, respectively. $\Gamma_{i}^{in}$ ($\Gamma_{i}^{out}$) is the set of upstream (downstream) neighbor nodes directly connecting to $v_{i}^{p}$.

If the load of node $v_{i}^{p}$ is less than the lower-bound capacity, the node will fail. If the load distribution of $v_{i}^{p}$ leads some neighboring nodes to fall below their capacity, this may trigger further failures in the neighboring nodes by load distribution. For example, if the node $v_{h}^{p}$ cannot sustain the load from $L_{v_{i}^{p}}$, this may result in failure of neighboring node $v_{s}^{p}$.

(10) $$L_{v_{i}^{p}}^{p}(0)-\Delta L_{is}^{p}{}^{-}<C_{v_{s}^{p}}(min)$$

Then, successive failures will occur on node $v_{e}^{p}$. This process continues until no failures occur and load redistributions in both networks finish [16].

#### 4.1.2. Overload Cascading Failure in the Cyber Network

When one node in a complex network fails, entirely or partially, and shifts its load to nearby nodes in the system, overloading could occur, leading to the failure of further nodes [36]. If this continues, overloading could fail the whole system. 

The cyber node failure will lead to larger-scale data collection and processing failures, so control signals will not be sent to the physical network in real time. In the cyber network, when a router fails, the untreated information will be redistributed to its neighboring nodes, and the information flow tends to choose routers with high processing capacity to maintain the smooth operation of the whole network [37]. Therefore, the load of the failed node will be redistributed to its nearest neighbor, depending on the probability:

(11) $$\Delta L_{is}^{c}{}^{+}=L_{v_{i}^{c}}(0)\frac{C_{v_{s}^{c}}-L_{v_{s}^{c}}}{\sum_{r\in \Gamma_{i}}\left(C_{v_{r}^{c}}-L_{v_{r}^{c}}\right)},\;$$

(12) $$\Delta L_{ih}^{c}{}^{+}=L_{v_{i}^{c}}(0)\frac{C_{v_{h}^{c}}-L_{v_{h}^{c}}}{\sum_{r\in \Gamma_{i}}\left(C_{v_{r}^{c}}-L_{v_{r}^{c}}\right)},\;$$

where $\Delta L_{is}^{c}{}^{+}$ and $\Delta L_{ih}^{c}{}^{+}$ are the increased load for $v_{s}^{c}$ and $v_{h}^{c}$, respectively, and $\Gamma_{i}$ is the set of neighboring nodes directly connecting to $v_{i}^{c}$.

If the load of $v_{s}^{c}$ and $v_{h}^{c}$ are more than the upper node capacity

(13) $$L_{v_{s}^{c}}(t)>C_{v_{s}^{c}},\;$$

(14) $$L_{v_{h}^{c}}(t)>C_{v_{h}^{c}},\;$$

then the successive failures will occur on $v_{s}^{c}$ and $v_{h}^{c}$ at time t, and its load will be redistributed to other functional nodes. All the remaining functional nodes will get an additional load from the failed nodes, which may lead to overloading and failure of the other nodes. The process takes place until no further failure is possible and the cyber supply network is considered stable.

#### 4.1.3. Cascading Failure in the Cyber-Physical Supply Networks

The cascading failure process in the cyber-physical supply networks can generally be described as follows [3,27,38]:

1. One or several nodes in cyber-physical supply networks will be selected as the initial failure. When a node fails, all connectivity links connected to it will become dysfunctional and are viewed as failed.
2. Load redistribution of failed nodes. In the physical supply network, failed nodes will reduce the loads of upstream and downstream nodes. In the cyber supply network, loads of failed nodes will redistribute to upstream and downstream nodes.
3. Calculate the new loads in the cyber-physical supply network. In the physical supply network, if the load of a node is less than its minimum capacity and the node fails, remove the underload nodes. In the cyber supply network, if the load of the nodes is more than its maximum capacity and the node fails, remove the overload nodes. For removed nodes, check whether the counterpart nodes fail or the neighbors fail.
4. A node that has no connections with a node from its couple network is regarded as failed. In the physical supply network, a node fails if its load is less than its minimum capacity. If the failed node is coupled to a counterpart node in the cyber supply network, then the counterpart node in the cyber supply network is also removed. In the cyber supply network, a node fails if its load is larger than its maximum capacity. If the failed node is coupled to a counterpart node in the physical supply network, then the counterpart node in the physical supply network is also removed.
5. Cascading failures will continue on the cyber-physical supply network until no further failed node and link occur.

**Figure 3:**
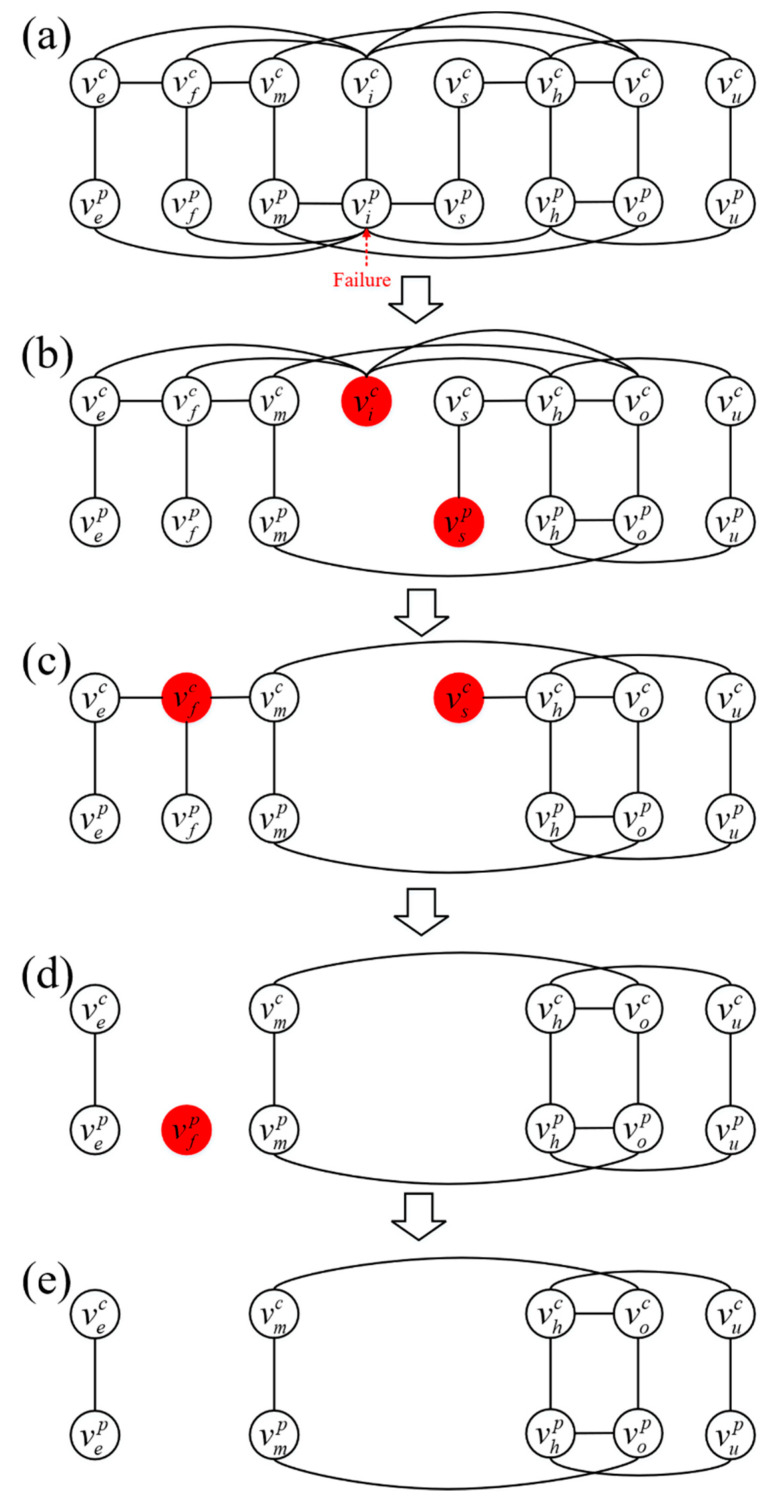
Illustration of a cascading failure that occurs in the cyber-physical supply networks under physical node failure.

As shown in Figure 3, a cascading failure is triggered by the failure of node $v_{i}^{p}$. First, $v_{i}^{p}$ is removed from the system along with its connectivity and dependency links. As a result, $v_{s}^{p}$ fails due to the load redistribution from $v_{i}^{p}$. The failure of $v_{i}^{p}$ means that $v_{i}^{s}$ has no support and consequently fails. Second, $v_{f}^{c}$ fails due to the load redistribution from $v_{i}^{c}$, and the failure of $v_{s}^{p}$ makes $v_{s}^{c}$ has no support and consequently fails. Third, $v_{f}^{p}$ fails from the lack of support due to the failure of node $v_{f}^{c}$. Finally, $v_{f}^{p}$ is removed, and the cascading failure stops. The remaining functioning nodes are $\left\{v_{e}^{p},v_{m}^{p},v_{h}^{p},v_{o}^{p},v_{u}^{p}\right\}$ in network $G^{p}$ and $\left\{v_{e}^{c},v_{m}^{c},v_{h}^{c},v_{o}^{c},v_{u}^{c}\right\}$ in network $G^{c}$.

### 4.2. Evaluation Index

Cascading failures can bring about significant degradation of performance. Several metrics have been developed to compute the damage caused by cascading failures, such as the size of the largest connected component [3,39] and the largest connected subgraph’s average degree [40]. We used the ratio of the survival nodes to measure the robustness of cyber-physical supply networks [41]:

(15) $$R=\frac{N_{p}^{\prime }+N_{c}^{\prime }}{N_{p}+N_{c}},\;$$

where $N_{p}$ and $N_{c}$ are the initial numbers of nodes in the physical supply network and cyber supply network, respectively. $N_{p}^{\prime }$ and $N_{c}^{\prime }$ are the numbers of nodes remaining in the network $G^{p}$ and $G^{c}$ after being attacked, respectively. 

## 5. Numerical Simulation

**Figure 4:**
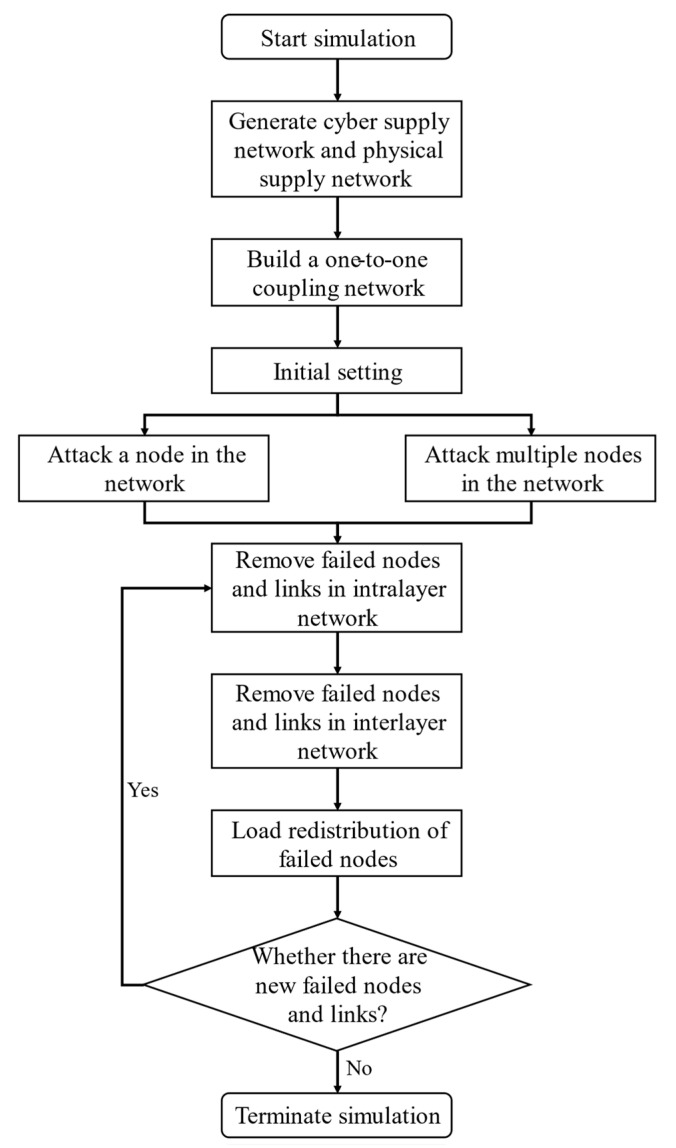
Flowchart of the numerical simulation.

We extracted the supply network data of China’s electric vehicle supply network as the physical supply network, and the cyber supply network as the BA scale-free network [16,42]. The physical supply network was obtained from a secondary data source using Mergent Online, which lists global firms’ information, including suppliers, customers, and competitors. The number of physical supply network nodes was 317, including 269 suppliers, five manufacturers, and 43 customers and the directed edges were 497. The cyber network had 317 nodes, and the average degree was about 5. The physical supply network and cyber supply network were fully coupled, meaning all nodes in the physical supply network were one-to-one coupled to nodes in the cyber supply network. All simulations were repeated 20 times to minimize randomness, and the average values were used for further analysis. A flowchart of the numerical simulation is presented in Figure 4 to intuitively describe the iterative procedure of cascading failure in cyber-physical supply networks.

### 5.1. The Cascading Process under Cyber Node Failure

#### 5.1.1. The Cascading Process of One Node Failure

**Figure 5:**
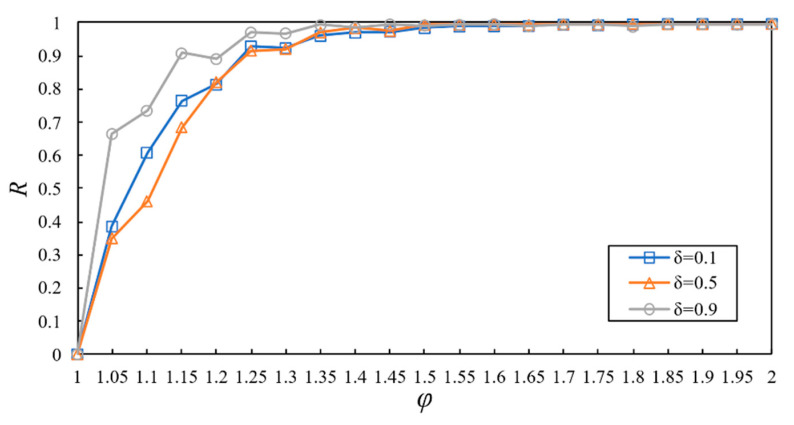
Relation between R and φ with different values of δ.

We first investigated the relationship between R and φ with different values of δ, and the results are presented in Figure 5. R ascends with the increase in φ. For the cyber supply network, nodes’ subsequent failures are provoked by loads exceeding the upper bound. The higher the upper-bound value, the smaller the cascading size [16]. R also ascends with the increase in δ. As a critical parameter for adjusting the cyber node capacity, the rise of δ can improve the network’s cascading robustness. For example, in the case of φ=1.1 and δ=0.1, R is 0.61. When φ stays the same, δ rises to 0.9, and R is 0.73, the cascading failures are reduced. Under the same set of other parameters, the increase of φ and δ can improve R, indicating that the damage of cascading failures to the network will decline. 

We also observed that there is a critical threshold $\varphi^{*}$. When φ is less than or equal to $\varphi^{*}$, the cascading failures can be triggered. When φ is greater than $\varphi^{*}$, the network will not have cascading failures. This is evident in the case of $\varphi^{*}=1.35$ and δ=0.9, and $\varphi <\varphi^{*}$ will lead to the cascading failures of the network. 

**Figure 6:**
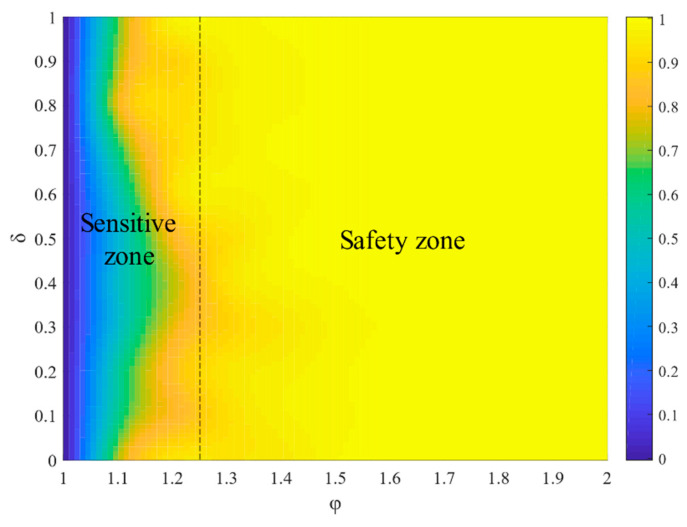
The effect of φ and δ on R.

Figure 6 presents the heatmaps of R within the parameter space [φ,δ]. We observed that the critical thresholds $\varphi^{*}$ and $\delta^{*}$ divide the heatmaps into two zones: safety zone and sensitive zone. In the safety zone, the value of R is relatively large, and R increases slightly with the increase in δ when φ>1.2. In the sensitive zone, the value of R is relatively small, and R increases significantly with the increase in δ when φ<1.2.

#### 5.1.2. The Cascading Process of Several Node Failures

**Figure 7:**
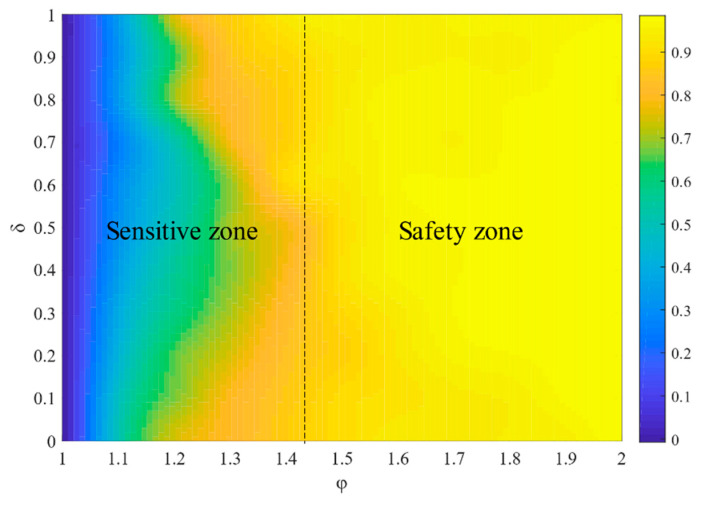
The effect of φ and δ on R under several node failures.

Figure 7 shows how the network robustness changes with φ and δ. The sizes of the safety zones become smaller under several node attacks. Parameter δ is much more influential, while the effect of φ is relatively weak, meaning that the node’s load in the cyber-physical networks has many effects on robustness.

**Figure 8:**
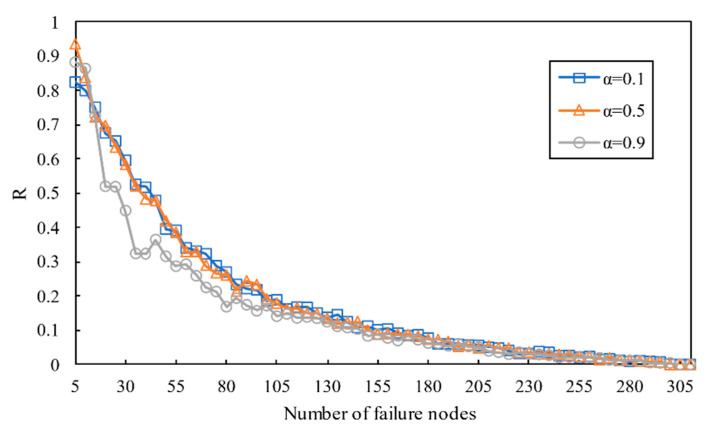
The relationship between robustness and the number of failure nodes with different values of α.

As shown in Figure 8, the influence of several node attacks on the robustness of networks is almost identical under different values of α, but α=0.9 harms networks more seriously than α=0.1 and α=0.5. With the increase in α, the networks become more sensitive to several node attacks.

### 5.2. The Cascading Process under Physical Node Failure

#### 5.2.1. The Cascading Process of One Node Failure

**Figure 9:**
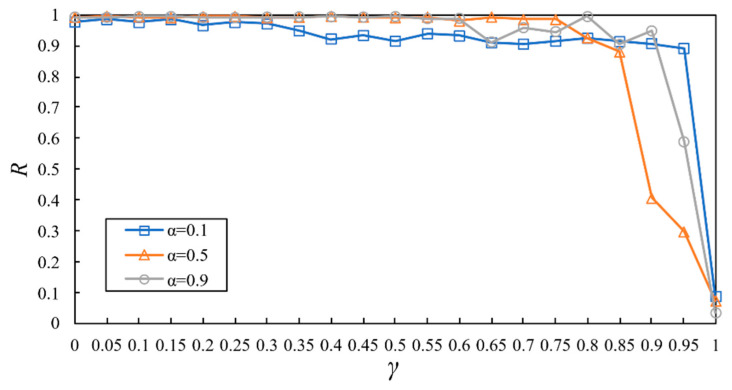
Relation between R and γ with different values of α.

Next, we investigated the relationship between R and γ with different values of α in cyber-physical supply networks, and the results are presented in Figure 9. First, as γ changes from 0 to 1, the robustness of the whole network decreases. R descends with the increase in γ. The reason is as follows: One firm’s failure can cause losses to its upstream and downstream firms, resulting in further failures of these firms. The successive failures of firms are caused by loads being less than the lower bound. A smaller γ could make the network more robust to cascading failure. The higher the value of the lower bound, the greater the cascading size [14]. 

Moreover, when the cyber-physical supply networks face node attacks, a critical threshold can determine whether the network will have cascading failures. When γ is less than or equal to $\gamma^{*}$, the network will not have cascading failures, and when γ is greater than $\gamma^{*}$, cascading failures can be triggered. As shown in Figure 9, when α=0.5, the critical threshold $\gamma^{*}=0.85$ and $\gamma >\gamma^{*}$ will lead to cascading failures in the network. The critical threshold is also observed in α. When α is less than $\alpha^{*}$, the increase of α is beneficial to the cascading robustness of the network. When α is greater than or equal to $\alpha^{*}$, the raising effect of node load expansion on cascading robustness will be saturated. 

**Figure 10:**
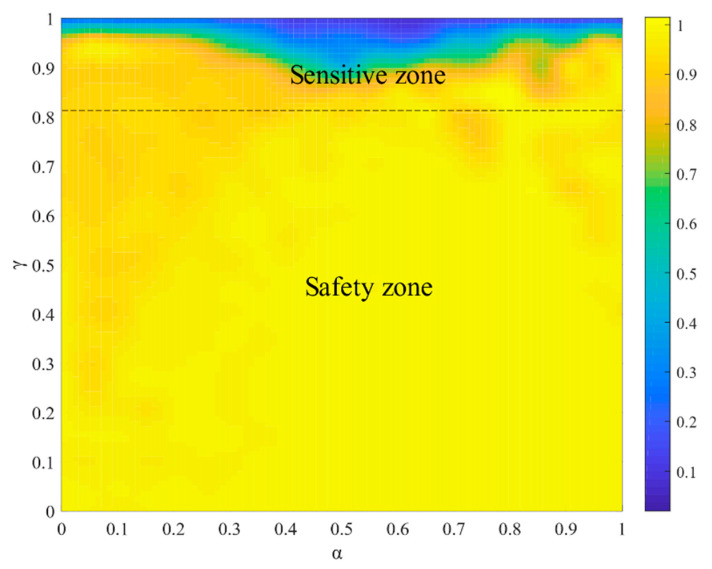
The effect of α and γ on R.

Figure 10 presents the heatmaps of R within the parameter space [α,γ]. The critical thresholds, $\alpha^{*}$ and $\gamma^{*}$, divide the heatmaps into two zones: the sensitive and safety zones. In the sensitive zone, the cascading robustness of the network varies with the setting of [α,γ]. When the capacity parameter space [α,γ] falls into the safety zone, the cascading robustness reaches the maximum value of 1, which means that cascading failure will not occur. The larger the safety zone, the lower the capacity extension costs to prevent cascading failures on the network [43].

#### 5.2.2. The Cascading Process of Several Node Failures

**Figure 11:**
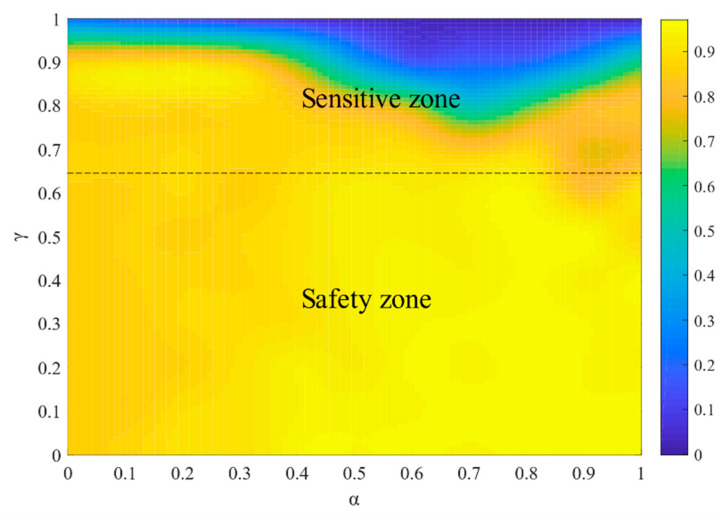
The effect of α and γ on R under several node failure.

In practical scenarios, several node attacks are also prevalent. This subsection evaluates the cascading robustness of the cyber-physical supply networks when facing several node attacks. Figure 11 shows the cascading robustness under several node attacks. We found that the sizes of safety zones become smaller under several node attacks. The parameter α is much more influential, while the effect of γ is relatively weak, meaning that the node’s capacity in the cyber-physical networks has many effects on robustness. 

**Figure 12:**
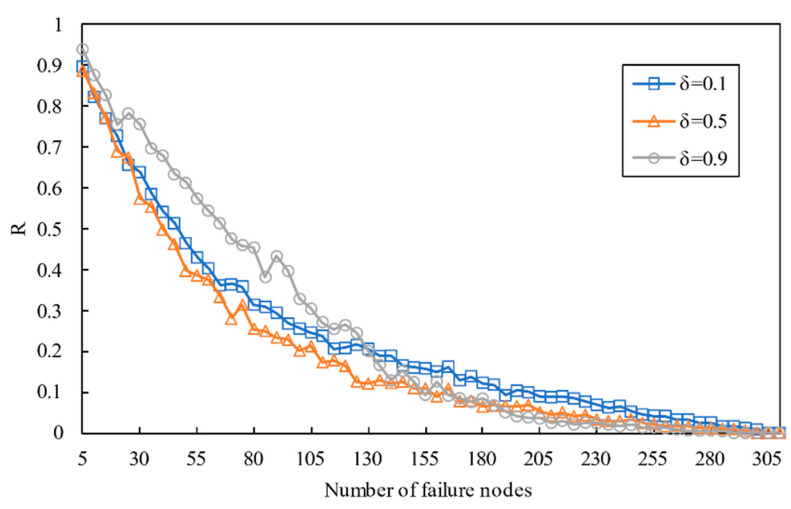
The relation between robustness and the number of failure nodes with different values of δ.

In Figure 12, with the increase in failure nodes, numerous nodes become faulty quickly. When a few fractions fall faulty, the damage caused by removing nodes is biggish. However, with the increased fraction of initial failure increased, the effect of damaging the network gradually decreases. Secondly, the cascading robustness ascends with the increase in δ. Due to the fact that the node load increases with an increase in δ, the number of failure nodes in the intralayer network and the couple network is more considerable. Thirdly, the process of cascading failure emerges a second-order transition.

## 6. Discussion and Conclusions

### 6.1. Discussion

The above results indicate that the occurrence of unexpected failures is sufficient to cause cascading failure across the whole cyber-physical supply network. The simulation results suggest several implications for managers to better deal with the mentioned failures and improve the robustness of the cyber-physical supply network.

Firms should improve robustness by establishing proper capacity. The allocated capacity is greater than the needed load to better withstand possible failures in the cyber-physical supply network. The experimental results display that the cascading failure depends on a specific capacity range, this could reduce the influence of node failure. Managers should properly allocate the capacity to resist failure and reduce the probability of failure; however, firm capacity is limited by the cost. Additionally, as the load increases, the strengthened capacity may gradually lose its effectiveness in mitigating cascading failures. Therefore, assigning a proper capacity in the cyber-physical supply network can not only assist in reducing the size of cascading failure, but also reduce the cost of maintaining the capacity.

The impact of cascading failures on the cyber-physical supply network should be comprehensively measured. Unlike the cascading failures in most physical supply networks resulting from overloading, this paper identified two kinds of cascading failure processes: underload cascading failure in the physical supply network and overload cascading failure in the cyber supply network. The proposed model can help managers to better understand the dynamic behaviors of cyber-physical supply networks during cascading failures. Therefore, to obtain relatively objective evaluation results, managers may need to measure the impact of cascading failures from different processes in the cyber-physical supply network.

This study is not without limitations. First, our physical supply network model was constructed from Mergent Horizon. The data were verified to be accurate and enable us to build China’s electric vehicle supply network. However, this dataset may not capture all the firms and relationships in the network. Second, our model concentrates on firms’ short-term reactions to a failure, as we delete firms from the cyber-physical supply network and do not consider if and when the firms will recover to regular operation. Lastly, we did not consider the adaptive strategies of firms confronted by cascading failures. In spite of these limitations, our study is very useful because this study uncovers the cascade window for cyber-physical supply networks, and the parameter space can determine the occurrence and scale of the cascading failure. 

### 6.2. Conclusions

We studied the robustness of the cyber-physical supply networks against cascading failures. Our model consists of a physical supply network where the failure of a node results in flow redistribution and possible further failures due to underloading, and a cyber supply network where the failure of a node leads to flow redistribution and possible further failures due to overloading. The relationship between the cyber supply network and physical supply is one-to-one interdependence. Besides, we employed China’s electric vehicle supply network and BA network to model cascading failures for cyber-physical supply networks. We obtained some meaningful results that can provide theoretical guidance to build cyber-physical supply networks with higher robustness:

Firstly, the negative correlation between the robustness of the cyber-physical supply networks and the upper-bound capacity parameter φ was proved in cyber node failure. In general, cyber devices do not operate at full capacity, and a larger value of φ means more redundant capacity of devices and can mitigate the cascading propagation.

Secondly, a positive relationship was observed between the lower-bound capacity and cascading size, and the size of cascading failures is mainly determined by γ under physical node failure. The lower-bound capacity parameter γ is related to the firm’s operating agility and resilience. A more competitive firm is often related to a lower value of γ.

Thirdly, a U-shaped relationship was observed between the node load and robustness of cyber-physical supply networks under cyber or physical node failure. As the load rises, the strengthened capacity may gradually lose its effectiveness in mitigating cascading failures, that is, as the load increases, it becomes increasingly difficult to reduce cascading failures by strengthening capacity.

In the future, we can develop cascade defense strategies to reduce the consequences of cascading failures in cyber-physical supply networks. Moreover, we will find the optimal cyber-physical supply networks with comprehensive tolerance against random and targeted failure.

## Figures and Tables

**Table 1:** Summary of the literature review.

Failure	Cascading Failure		Supply Network Domain		Robustness Metrics	Work
Node Failure	Overload	Underload	Cyber Supply Network	Physical Supply Network		
✓	✓		✓	✓	Comprehensive effectiveness index	[15]
✓	✓		✓	✓	Network efficiency and percentage of unserved nodes	[16]
✓	✓		✓	✓	Fraction of surviving nodes	[17]
✓	✓			✓	Number of surviving nodes	[18]
✓		✓		✓	Network efficiency	[14]
✓		✓		✓	Network efficiency	[19]
✓		✓		✓	Fraction of failed nodes	[13]
✓	✓			✓	Dynamic network load entropy	[20]
✓	✓	✓	✓	✓	Number of surviving nodes	This work

**Table 2:** Symbols used in this paper and their corresponding meanings.

Notation	Meaning
$G^{p}$	Physical supply network
$G^{c}$	Cyber supply network
$V^{p}$	Set of nodes in the physical supply network
$V^{c}$	Set of nodes in the cyber supply network
$v_{i}^{p}$	A node i in the physical supply network
$v_{i}^{c}$	A node i in the cyber supply network
$E^{p}$	Set of links in the physical supply network
$e_{ij}^{p}$	A directed connection from node $v_{i}^{p}$ to node $v_{j}^{p}$ in the physical supply network
$e_{ij}^{c}$	A connection from node $v_{i}^{c}$ to node $v_{j}^{c}$ in the cyber supply network
$E^{c}$	Set of links in the cyber supply network
$E_{ik}^{pc}$	Set of dependency links connecting nodes between network $G^{p}$ and network $G^{c}$
$W^{p}$	A weighted adjacency matrix of physical supply network
$w_{ij}^{p}$	The weight of the link $e_{ij}^{p}$
$W^{c}$	A weighted adjacency matrix of the cyber supply network
$w_{ij}^{c}$	Weight of link $e_{ij}^{c}$
α	Tunable parameter used to adjust the initial load in the physical supply network
β	Upper-bound capacity parameter of the node in the physical supply network
γ	Lower-bound capacity parameter of the node in the physical supply network
θ	Weight parameter of the link in the physical supply network
$k_{v_{i}^{p}}$	Degree for node $v_{i}^{p}$
$k_{v_{i}^{p}}^{in}$	In-degree of the node $v_{i}^{p}$
$k_{v_{i}^{p}}^{out}$	Out-degree of the node $v_{i}^{p}$
$L_{v_{i}^{p}}(t)$	Load for node $v_{i}^{p}$ attime t
$L_{v_{i}^{c}}(t)$	Load for node $v_{i}^{c}$ at time t
$C_{v_{i}^{p}}$	Capacity for node $v_{i}^{p}$
$C_{v_{i}^{c}}$	Capacity for node $v_{i}^{c}$
δ	Tunable parameter used to adjust the initial load in the cyber supply network
φ	Upper-bound capacity parameter of the node in the cyber supply network
τ	Weight parameter of the link in the cyber supply network

## Data Availability

The data used to support the findings of this study are available from the corresponding author upon request.
